# Laparoscopy in the Treatment of Early Cervical Carcinoma

**DOI:** 10.1155/DTE.1.19

**Published:** 1994

**Authors:** Alton V. Hallum, III, Joel M. Childers

**Affiliations:** 1 Department of Obstetrics and Gynecology Division of Gynecologic Oncology University of Arizona USA; 2 2625 N. Craycroft Road Suite 201 Tucson Arizona 85712 USA

## Abstract

Recent investigators have used several indications to incorporate laparoscopy in the management of patients with cervical cancer. This manuscript reviews the current literature on the role of modern operative laparoscopy in early cervical cancer and recommends a simple approach for its use in these patients.


*Diagnostic and Therapeutic Endoscopy*, Vol. 1, pp. 19–23
Reprints available directly from the publisher
Photocopying permitted by license only

© 1994 Harwood Academic Publishers GmbH
Printed in Malaysia

### Laparoscopy in the Treatment of Early Cervical Carcinoma

ALTON V. HALLUM, III[1] and JOEL M. CHILDERS[2]

[1]*Assistant Professor of Clinical Obstetrics and Gynecology,* [2]*Assistant Professor of Clinical Obstetrics and Gynecology Department of Obstetrics and Gynecology, Division of Gynecologic Oncology, University of Arizona*

*(Received February 8, 1994; in final form February 10, 1994)*

Recent investigators have used several indications to incorporate laparoscopy in the management of patients with cervical cancer. This manuscript reviews the current literature on the role of modern operative laparoscopy in early cervical cancer and recommends a simple approach for its use in these patients.

**KEY WORDS:** Laparoscopy-cervical cancer, Laparoscopic lymphadenectomy, Laparoscopic radical hysterectomy

### INTRODUCTION

Prior to 1987, the role of operative laparoscopy in patients with gynecologic malignancies was very limited. Interest was revived among gynecologic oncologists with the development of videolaparoscopy and sophisticated instrumentation. These advances allow gynecologic surgeons to explore and operate in the retroperitoneal space. Recently, several groups of investigators have incorporated this technique into the surgical treatment of early cervical cancer. Benefits include shorter hospitalization, decreased morbidity, and avoidance of laparotomy in patients with metastatic disease. In addition, radical vaginal hysterectomy can be performed in conjunction with laparoscopic pelvic and paraaortic lymphadenectomy. While the reports are few, the results they present are promising and supply a sound foundation for future studies of this new approach to early cervical cancer. This manuscript will review how laparoscopy has uses both in the lymphadenectomy and in the radical hysterectomy. Table 1 outlines the potential uses of laparoscopy in patients with early cervical cancer.

### TRADITIONAL MANAGEMENT OF CERVICAL CANCER

Traditionally, because of the inaccuracies of clinical staging (LaPolla *et al.*, 1986), the treatment of patients with early cervical cancer has involved a laparotomy to search for metastatic disease. If there is no obvious spread of the tumor at the time of surgery, pelvic and paraaortic lymph nodes are removed and suspicious nodes evaluated by frozen section. Lymph node dissection is warranted to determine which patients will benefit from radical surgery. A radical hysterectomy is performed if the nodes do not contain metastases. The type of radical hysterectomy as well as the completeness of lymphadenectomy vary depending on the surgeon's philosophy and the tumor size. When lymph node metastases occur, the number and location of positive nodes as well as the surgeon's bias dictate whether the radical hysterectomy is completed or aborted (Potter *et al.*, 1990). Patients with positive nodes who require radiation therapy are at increased risk of experiencing treatment complications if they have had recent surgery (Weiser *et al.*, 1989).

### LAPAROSCOPIC LYMPHADENECTOMY

The French have been instrumental in pioneering endoscopic approaches to the retroperitoneal space. Daniel

**Table 1** Potential Uses of Laparoscopy in Early Cervical Cancer

- Evaluate / Sample Lymph Nodes
  - Extraperitoneal
  - Transperitoneal
- Convert Radical Abdominal Hysterectomy to Radical Vaginal Hysterectomy
  - Laparoscopic Assisted Radical Vaginal Hysterectomy (Celio-Schauta)
  - Laparoscopic Radical Hysterectomy

Address for correspondence: Joel M. Childers, M.D., 2625 N. Craycroft Road, Suite 201, Tucson, Arizona 85712.

Dargent (Dargent, 1987) was the first to report an endoscopic technique to remove pelvic lymph nodes in patients with early cervical cancer. In 1987 he described his experience combining laparoscopic retroperitoneal pelvic lymphadenectomy with radical hysterectomy in these patients (Dargent, 1987). His approach involves insertion of the primary laparoscopic trocar into the suprapubic preperitoneal space and is assisted by two ancillary trocars introduced into the inguinal and paramedian infraumbilical areas. Using this approach, he was able to extract the lymphatic chain over the external iliac vessels up to the bifurcation of the common iliac artery. These nodes, as well as those lying between the iliac vein and the obturator nerve, were removed for histologic analysis. Of six patients in this preliminary study who immediately underwent laparotomy, none had positive lymph nodes which were not identifed laparoscopically. The author concluded that the procedure was both safe and accurate in the assessment of these patients.

Querleu (Querleu, *et al.*, 1991) subsequently described a transperitoneal approach to sampling the pelvic lymph nodes. The procedure entails placement of four laparoscopic ports, two in the midline and two laterally. The pelvic peritioneum is incised parallel to the external iliac vessels between the round and infundibulopelvic ligaments to allow access to the retroperitoneal space. The targeted pelvic lymph nodes are then identified and removed for histologic analyses in a manner that is akin to the method used at laparotomy.

Querleu (Querleu, *et al.*, 1991) utilized this laparoscopic staging technique on 39 patients with FIGO stages IB-IIB cervical cancer. In two patients, needle aspiration of unresectable nodes confirmed the presence of metastases. Three additional patients, who had positive lymph nodes detected at laparoscopy avoided further surgery. Of the remaining 34 patients, 2 underwent radical vaginal hysterectomy and 32 underwent radical abdominal hysterectomy with removal of all remaining lymph node tissue. Among the 32 patients who were treated with radical abdominal hysterectomy, no lymph node metastases were detected at laparotomy. However, in a later report Querleu (Querleu, 1993) details the case of a patient in whom laparoscopic pelvic node dissection was negative but in whom a node resected at the time of radical hysterectomy was positive.

The first American series in which laparoscopic lymphadenectomy was used in the management of cervical cancer was reported by Childers and colleagues (Childers *et al.*, 1992) at the University of Arizona. This study reported their experience with 18 patients with both early and advanced cervical cancer. Eight patients with stage IB tumors underwent laparoscopic staging prior to planned radical hysterectomy. At laparoscopy, 3 of these 8 patients were found to have positive lymph node metastases and the planned radical hysterectomy was aborted. The 5 remaining patients underwent radical abdominal hysterectomies with removal of all remaining lymph node tissue. In 3 of these 5 patients, no residual lymph nodes were found. In 1 of the 5 patients, 3 nodes, without metastases, were detected at the time of radical hysterectomy. In the remaining case, 11 pelvic lymph nodes were removed during laparotomy. This patient had 1 parameterial node positive for microscopic disease. The 3 patients in whom all the lymph nodes were resected laparoscopically weighed 120 pounds or less. The patient with 3 remaining nodes weighed 160 pounds, while the patient with 11 remaining nodes weighed more than 180 pounds. Overall 91% of the nodes normally taken at the time of radical hysterectomy were removed laparoscopically. While the numbers are small, this data suggests that patient habitus influences the ability, especially of the novice laparoscopist, to complete a thorough laparoscopic lymph node dissection.

To determine the feasibility and safety of a laparoscopic paraaortic lymphadenectomy, the same investigators also removed the paraaortic nodes in 16 of their patients. This additional dissection was performed in patients with both early and advanced disease. Microscopic involvement of the paraaortic nodes was found in 1 patient who had gross involvement of the pelvic lymph nodes. Skip metastases to the paraaortic chain were not detected and they reported no complications.

Nezhat (Nezhat *et al.*, 1992) published his initial experience with combined laparoscopic pelvic and paraaortic lymph node dissection in a patient with stage IA2 cervical cancer. No positive nodes were found. Subsequently, he extended his series to include 18 patients with stage IA2 (10 patients), IB (7 patients) and IIA (1 patient) disease (Nezhat *et al.*, 1993). Paraaortic lymph node samplings were performed in 3 stage IA2 patients who had lymphvascular involvement and in all the higher stage patients. Eleven of 18 patients underwent paraaortic lymphadenectomies with a median of 5 nodes sampled (range 3–9). They also reported no complications from paraaortic lymph node sampling.

Querleu (Querleu *et al.*, 1993) described 4 patients, 2 with cervical and 2 with ovarian cancer, in whom paraaortic lymph node samplings were performed. One patient with cervical cancer, who had previously undergone laparoscopic staging with pelvic lymph node dissection, had an unexpected nodal metastasis discovered at the time of radical hysterectomy. A second laparoscopy was performed to sample the paraaortic nodes. The second patient underwent paraaortic node dissection as part of her original procedure. Between 6–9 paraaortic nodes were sampled in each patient without complication, and all were negative for metastatic disease.

Fowler (Fowler, *et al.*, 1993) at the University of Minnesota, has reported 12 patients with stage IB cervix cancer who underwent a laparoscopic, transperitoneal lymphadenectomy followed immediately by laparotomy. The yield of lymph nodes obtained laparoscopically was 75%; 282 nodes were obtained at laparoscopy and 95 at laparotomy. Interestingly, a clear improvement in yield was evident as the operators gained experience with the technique; the percentage of lymph nodes removed through the laparoscope increased from 63% in the first 6 patients to 85% in the last 6 patients. Nodes containing metastatic disease were found in 2 patients. In both cases, these nodes were removed laparoscopically. No further nodal metastases were detected at laparotomy. He also performed right-sided paraaortic lymph node samplings in 2 of 12 patients who went on to laparotomy. One patient had a grossly positive pelvic node metastasis at the time of laparoscopy; involving 2 of 4 paraaortic nodes disected. The second patient had neither pelvic nor paraaortic metastases. No residual right-sided paraaortic nodes were detected in these patients at laparotomy.

Initial reports indicate that laparoscopic lymphadenectomy is an attractive and acceptable alternative to laparotomy in the evaluation of patients prior to planned radical hysterectomy. Few complications have been reported in the patients undergoing laparoscopic pelvic and paraaortic lymph node dissections. The estimated blood loss was less than 100 cc in all cases in which it was reported (Childers *et al.*, 1992), and the procedure were performed in a timely fashion, from 2 to 2.5 hours on average (Childers *et al.*, 1992; Nezhat *et al.*, 1993). The average length of hospital stay has been short, in Childers' study, 1.5 days, in Querleu's study, 2 days. Furthermore, the recovery from surgery was rapid; patients staged laparoscopically were able to eat the day of their procedure (Childers *et al.*, 1992). Overall, the adequacy of laparoscopic lymph node resection, combining data from the studies in which radical hysterectomy followed laparoscopic staging, appears to be acceptable. The visual inspection of disection sites and node counts are comparable to laparotomy.

### LAPAROSCOPIC ROLE IN RADICAL HYSTERECTOMY

The laparoscope also has permitted adaptations in the approach to radical hysterectomy in patients with early cervical cancer. Laparoscopic radical hysterectomy was described by both Canis (Canis *et al.*, 1990; Canis *et al.*, 1992) and Nezhat (Nezhat *et al.*, 1992; Nezhat *et al.*, 1993). This approach, while theoretically sound, has met with two major difficulties. First, the procedure is time—consuming, from 4–8 hours in Nezhart's series of 7 patients. Second and more importantly, the radical nature of the procedure has been questioned (Dargent 1993; Spirtos, 1993). One of the authors commented that a type II (modified) radical hysterectomy was performed (Burrell, International Gynecologic Cancer Society Workshop, 1993).

Laparoscopically assisted radical vaginal hysterectomy was described by Querleu (Querleu 1991; Querleu, 1993) and by Dargent (Dargent, Mathevet, 1992). In his later report, Querleu apportioned the laparoscopic and vaginal parts of the operation according to the type of radical hysterectomy. During type 2 or 3 radical hysterectomies, the lymphadenectomy and ureteral dissections were performed laparoscopically, while the parametria were divided via a vaginal approach. Perineal incisions were not required. On average, the procedures took approximately 4.5 hours to complete. Complications were uncommon, 2 patients required blood transfusions for blood loss estimated to be greater than 1000 cc, no unscheduled laparotomies were performed. The average hospital stay was 4 days. Querleu provides 1–3 year follow up information on the patients he describes. All but one remain disease—free, one patient required a posterior exenteration for a recurrence involving the rectum.

Dargent prefers the laparoscopic assisted radical vaginal hysterectomy, which he terms the "celio-Schauta." In contrast to Querleu, he transects the parametria laparoscopically and disects the ureters vaginally. An endo GIA stapler is used to divide the parametria at the pelvic sidewall. Shuchart incisions are usually avoided but are used when additional exposure is necessary. The advantage with this approach expressed by Dargent (Dargent, personal communication) is maintaining the radicality of the procedure, while drastically reducing the operative time. He has also performed a traditional radical vaginal hysterectomy, proceeded by negative laparoscopic pelvic lymph node dissection (Dargent 1993, Dargent, 1993). The time required to complete the procedure averaged 1.5 hours. The long-term outcomes matched those achieved with abdominal surgery.

Currently, we would recommend the following approach to patients with cervical cancer stages IA2, IB, IIA, and IIB (Fig. 1): firstly, laparoscopic pelvic node dissection should be performed via either the transperitoneal or retroperitoneal approach. Patients with negative nodes can then be managed either with a laparoscopically assisted radical vaginal hysterectomy, a radical vaginal hysterectomy, or an abdominal radical hysterectomy. When pelvic node metas-

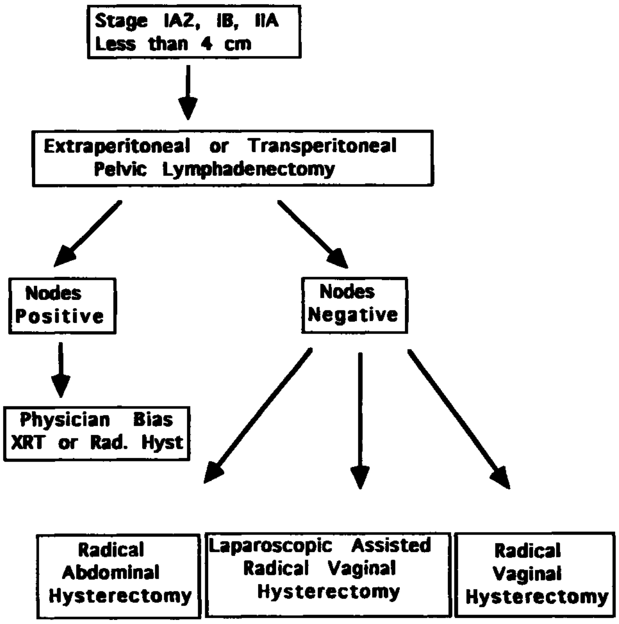

**Figure 1** Laparoscopy and cervical cancer Algorithm.

tases are discovered, we then evaluate the paraaortic nodes in order to select candidates who might benefit from extended field radiation therapy. Whether one proceeds with radical abdominal hysterectomy in the face of 1 or 2 positive nodes depends on the surgeon's philosophy.

These initial reports are promising and merit continued investigation. We believe that laparoscopic lymphadenectomy is a safe, feasible, and thorough procedure. Advantages include short hospitalization, low morbidity, avoidance of laparotomy, and individualization of therapy. Furthermore, in combination with laparoscopic lymph node dissection, surgeons can reasonably consider radical vaginal hysterectomy. The reemergence of the radical vaginal hysterectomy may be the greatest contribution of laparoscopy to the management of early cervical cancer. Learning these laparoscopic procedures is humbling and time-consuming. A steadfast and long-term commitment is necessary to gain expertise. Follow up reports of patients treated with these new techniques are necessary to ensure that survival is not compromised by these laparoscopic modifications of traditional surgery.

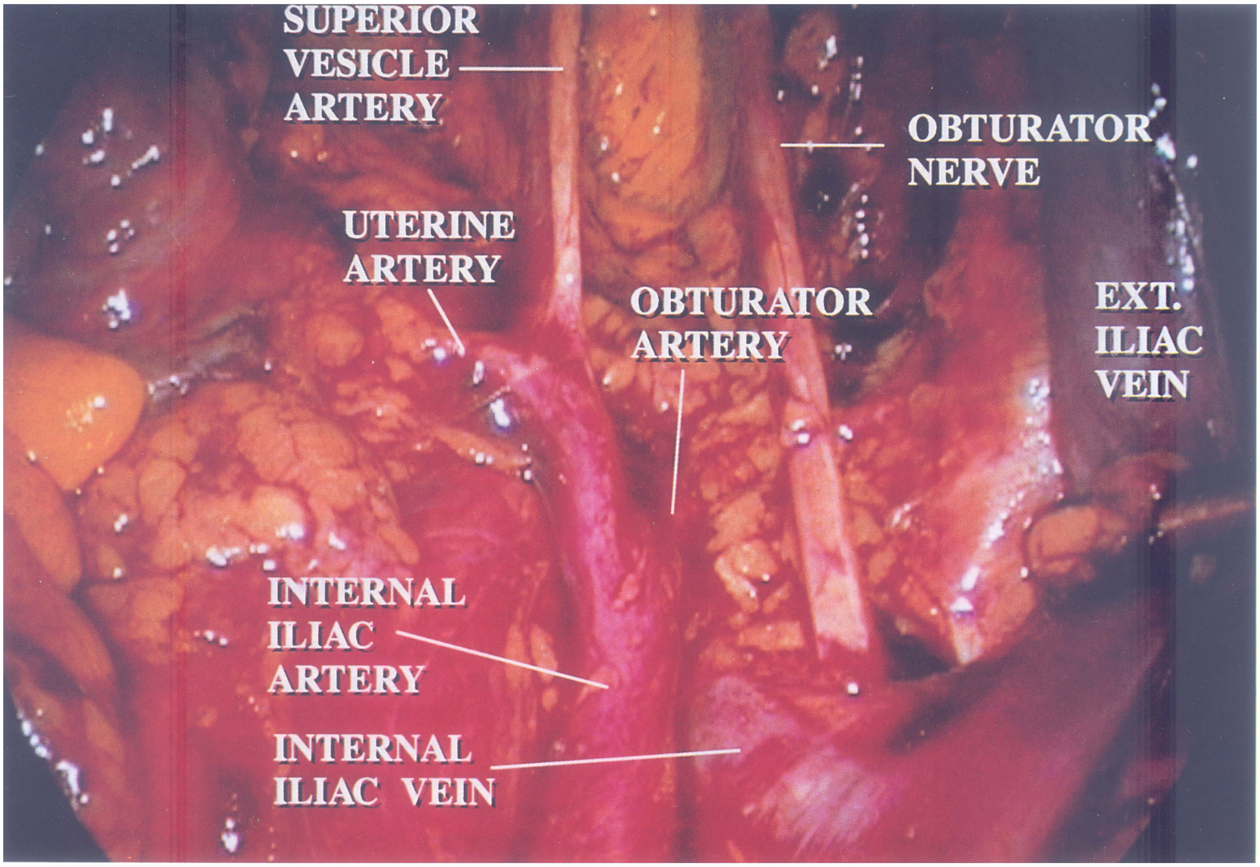

**Figure 2** This figure illustrates the right pelvis after a laparoscopic pelvic lymphadenectomy. The right external iliac artery has been retracted laterally to demonstrate the bifurcation of the iliac vein.

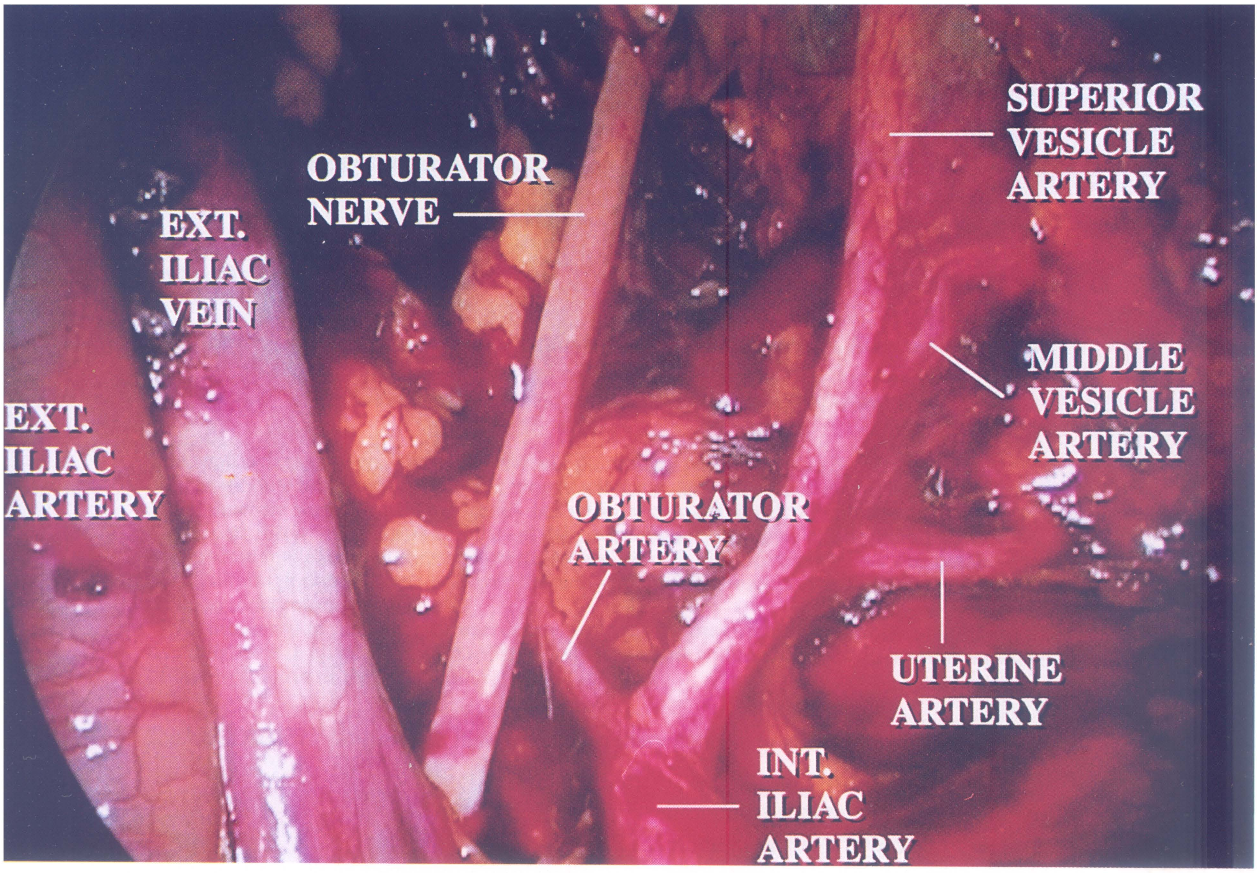

**Figure 3** This figure illustrates the lower left pelvis after a laparoscopic pelvic lymphadenectomy.

### REFERENCES

Burrell, M.: Fourth Biennial Meeting of the International Gynecologic Cancer Society. Stockholm Sweden, September 1, 1993

Canis M., Mage G., Wattiez A., *et al.*: Vaginally assisted laparoscopic radical hysterectomy. *J Gynecol Surg*, 1992;8:103–105

Childers J. M., Hatch K., Surwit E. A.: The role of laparoscopic lymphadenectomy in the management of cervical carcinoma. *Gynecologic Oncology*, 1992;47:38–43

Dargent D., Arnould P.: Percutaneous pelvic lymphadenectomy under laparoscopic guidance. In: Nichols D. (ed.): *Gynecologic and Obstetric Surgery*, Mosby Yearbook, Philadelphia, 1993

Dargent D., Mathevet P.: Hysterectomie elargie laparoscopico-vaginale. *J Gynecol Obstet Biol Reprod*, 1992;21:709–710

Dargent D., Roy M., Keita N., *et al.*: The Schauta operation: its place in the management of cervical cancer in 1993. 24th Annual Meeting The Society of Gynecologic Oncolgists, 1993; abstract 11

Dargent D.: A new future for Shauta's operation through pre-surgical retroperitoneal pelviscopy. *Eur J Gynecol Oncol*, 1987;8:292–296

Dargent, D.: Laparoscopic surgery and gynecologic cancer. *Current Opinion Obstet Gynecol*, 1993;5:294–300

Fowler J. M., Carter J. R., Carlson J. W., *et al.*: Lymph node yield from laparoscopic lymphadenectomy in cervical cancer: a comparative study. *Gynecologic Oncology*, 1993;51:187–192

La Polla J. P., Schlaerth J. B., Gaddis O., *et al.*: Influence of surgical staging on the evaluation and treatment of patients with cervical carcinoma. *Gynecologic Oncology*, 1986;24:194–206

Nezhat C. R., Burrell M. O., Nezhat F. R., *et al.*: Laparoscopic radical hysterectomy with paraaortic and pelvic node dissection. *Am J Obstet Gynecol*, 1992;166:864–865

Nezhat C. R., Nezhat F. R., Burrell M. O., *et al.*: Laparoscopic radical hysterectomy and laparoscopically assisted vaginal radical hysterectomy with pelvic and paraaortic lymph node dissection. *J Gynecologic Surgery*, 1993;9:105–120

Potter M. E., Alvarez R. D., Shingleton H. M., *et al.*: Early invasive cervical cancer with pelvic lymph node involvement: To complete or not to complete radical hysterectomy. *Gynecologic Oncology*, 1990;37:78–81

Querleu D., Leblanc E., Castelain B.: Laparoscopic pelvic lymphadenectomy in the staging of early carcinoma of the cervix. *Am J Obstet Gynecol*, 1991;164:579–581

Querleu D.: Hysterectomies elargies de Schauta-Amreich et Schauta-Stoechel assistees par coelioscopie. *J Gynecol Obstet Biol Reprod*, 1991;20:747–748

Querleu D.: Laparoscopic paraaortic lymph node sampling in gynecologic oncology: A preliminary experience. *Gynecologic Oncology*, 1993;49:24–29

Querleu D.: Laparoscopically assisted radical vaginal hysterectomy. *Gynecologic Oncology*, 1993;51:248–254

Spirtos N. M.: Laparoscopic radical hysterectomy with paraaortic and pelvic lymph node dissection? *Am J Obstet Gynecol* (letter), 1993;168:1643

Weiser E. B., Bundy B. N., Hoskins W. J., *et al.*: Extraperitoneal versus transperitoneal selective lymphadenectomy in the pretreatment surgical staging of advanced cervical cancer (A GOG study). *Gynecologic Oncology*, 1989;33:283–289

